# Piceatannol Increases Antioxidant Defense and Reduces Cell Death in Human Periodontal Ligament Fibroblast under Oxidative Stress

**DOI:** 10.3390/antiox9010016

**Published:** 2019-12-23

**Authors:** Flávia Póvoa da Costa, Bruna Puty, Lygia S. Nogueira, Geovanni Pereira Mitre, Sávio Monteiro dos Santos, Bruno José Brito Teixeira, Maria Sueli da Silva Kataoka, Manoela Domingues Martins, Carlos Augusto Galvão Barboza, Marta Chagas Monteiro, Hervé Rogez, Edivaldo Herculano Corrêa de Oliveira, Rafael Rodrigues Lima

**Affiliations:** 1 Laboratory of Functional and Structural Biology, Institute of Biological Sciences, Federal University of Pará (UFPA), Belém-Pará 66075-110, Brazil; fefcosta22@gmail.com (F.P.d.C.); brunaputy@gmail.com (B.P.); nogueiralygia@gmail.com (L.S.N.); 2 Laboratory of Tissue Culture and Cytogenetics, Environment Section, Evandro Chagas Institute, Ananindeua-Pará 67030-000, Brazil; ehco@ufpa.br; 3 Laboratory of Cell Culture, Faculty of Dentistry, Federal University of Pará (UFPA), Belém-Pará 66075-110, Brazil; geovannimitre@gmail.com (G.P.M.); sukataoka@yahoo.com.br (M.S.d.S.K.); 4 Laboratory of Oxidative Stress and Clinical Immunology, Faculty of Pharmacy, Federal University of Pará (UFPA), Belém-Pará 66075-110, Brazil; saviomontsan@gmail.com (S.M.d.S.); martachagas2@yahoo.com.br (M.C.M.); 5 Center for Valorization of Amazonian Bioactive Compounds (CVACBA) & Federal University of Pará UFPA, Belém-Pará 66075-110, Brazil; bjbteixeira@gmail.com (B.J.B.T.); herverogez@gmail.com (H.R.); 6 Department of Oral Pathology, School of Dentistry, Federal University of Rio Grande do Sul, Porto Alegre 91509-900, RS, Brazil; manomartins@gmail.com; 7 Department of Morphology, Federal University of Rio Grande do Norte, Natal 59078-970, Brazil; cbarbozag@yahoo.com

**Keywords:** stilbenes, viability, hydrogen peroxide, piceatannol, periodontal ligament

## Abstract

Piceatannol is a resveratrol metabolite that is considered a potent antioxidant and cytoprotector because of its high capacity to chelate/sequester reactive oxygen species. In pathogenesis of periodontal diseases, the imbalance of reactive oxygen species is closely related to the disorder in the cells and may cause changes in cellular metabolism and mitochondrial activity, which is implicated in oxidative stress status or even in cell death. In this way, this study aimed to evaluate piceatannol as cytoprotector in culture of human periodontal ligament fibroblasts through in vitro analyses of cell viability and oxidative stress parameters after oxidative stress induced as an injury simulator. Fibroblasts were seeded and divided into the following study groups: control, vehicle, control piceatannol, H_2_O_2_ exposure, and H_2_O_2_ exposure combined with the maintenance in piceatannol ranging from 0.1 to 20 μM. The parameters analyzed following exposure were cell viability by trypan blue exclusion test, general metabolism status by the 3-[4,5-dimethylthiazole-2-yl]-2,5-diphenyltetrazolium bromide (MTT) method, mitochondrial activity through the ATP production, total antioxidant capacity, and reduced gluthatione. Piceatannol was shown to be cytoprotective due the maintenance of cell viability between 1 and 10 μM even in the presence of H_2_O_2_. In a concentration of 0.1 μM piceatannol decreased significantly cell viability but increased cellular metabolism and antioxidant capacity of the fibroblasts. On the other hand, the fibroblasts treated with piceatannol at 1 μM presented low metabolism and antioxidant capacity. However, piceatannol did not protect cells from mitochondrial damage as measured by ATP production. In summary, piceatannol is a potent antioxidant in low concentrations with cytoprotective capacity, but it does not prevent all damage caused by hydrogen peroxide.

## 1. Introduction

Phytotherapy represents a science-based medical practice that use of plants either to treat disease or as health-promoting agents. It has been gaining importance in health field based specially in its anti-microbiological, anti-inflammatory, antioxidant, and antitumor properties [1]. In dentistry, several phytotherapeutic agents like curcumin, bromelain, chamomilla, and baicalin among others have been used especially in inflammatory conditions because they can modulate the inflammatory process, reduce pain, and promote faster wound healing with excellent clinical response [2,3,4,5,6,7]. 

Oxidative stress is a significant process in oral mucosal disease pathogenesis [8,9]. It represents the imbalance between the production of free radicals and the ability of the body to eliminate these reactive species. Reactive oxygen species (ROS) are the most important free radicals generated with biological beneficial effects at low level and normal conditions. However, higher concentrations of ROS become prejudicial to the organism causing cellular damage [10], resulting in changes in cellular metabolism, mitochondrial activity, and damage on proteins, lipids, and DNA. ROS generation is counterbalanced by the action of antioxidant mechanisms act as free radical scavengers and neutralize excess of ROS. The antioxidant system comprises several enzymatic and non-enzymatic components such as superoxide dismutase (SOD), glutathione peroxidase (GPx), catalase, carotenoids, phenolic acids, flavonoids, tannins, and others [9,11]. Some studies have shown the beneficial role of phytotherapic formulations against oxidative stress-related human diseases, including periodontal diseases; however, the mechanism involved in the positive response is not completely understood [12,13]. 

The stilbene piceatannol (3,3′,4,5′-tetrahydroxy-trans-stilbene), a resveratrol (trans-3,5,4′-trihydroxyestilbene) metabolite was first isolated from the plant Euphorbia lagasacae Spreng 1821 (WCSP, 2018) in 1984 [14] and has been found in different sources such as grapes, peanuts, sugar cane, blueberries, and passion fruit seeds [15]. Piceatannol has potent biological activities, including antioxidant, anti-cancer, anti-inflammatory, and anti-obesity properties. Interestingly, in comparison with red grapes, a major source of piceatannol in the human diet, the passion fruit representative sample contained 1000 to 2000 times more piceatannol in fresh matter terms. The passion fruit appears therefore as a new and promising source of piceatannol, which can be used as material for the production of nutraceuticals. Recent studies showed the piceatannol superiority over the resveratrol as antioxidant and cytoprotector [16] due to its higher capacity of chelators/sequestrants reactive oxygen species (ROS) [17]. Maternal supplementation with piceatannol is neuroprotective in rat neonatal hypoxia-ischemia [18]. Piceatannol affects peripheral clock gene expression and may prevent circadian disturbance [19].

Periodontal diseases usually refer to common inflammatory disorders, known as gingivitis and periodontitis, that involve a multifactorial interaction among microbial, host immunological response, and environmental modulating factors. High levels of nuclear factor-kappa B (NF-κB), transglutaminase 2 (TG2) and several inflammatory mediators, including IL-1, IL-6, TNF-α, and prostaglandin E2 are produced in periodontal diseases [20]. These processes result in the destruction of the tissues surrounding and supporting the teeth, bone resorption, and tooth loss [21]. Accumulating evidence has indicated a close connection between oxidative stresses in the pathogenesis of periodontal diseases. Inflammatory conditions that affect the periodontal ligament have been associated to an increase in ROS production that results in periodontal ligament destruction, bone resorption activity by stimulation of osteoclastogenesis, and decreased differentiation of osteoblasts [22]. In addition, periodontal treatment reduces inflammation and may be beneficial for periodontitis patients’ systemic and local oxidative stress control [23,24,25]. 

Thus, there is a therapeutic need to increase the defenses in the periodontal tissues and various compounds with antioxidant action are being tested [26]. Adjunctive local phytotherapy has been used in periodontal diseases and has been associated with significant improvement in periodontal clinical parameters (PPD and clinical attachment level) of patients [27]. In this way, resveratrol has demonstrated an antioxidant effect on gingival fibroblasts and in prevention of periodontal diseases progression in pre-clinical studies [28]. Piceatannol is superior to resveratrol concerning the antioxidant and cytoprotecting effects [16]. Therefore, the present study aimed to evaluate the piceatannol cytoprotection in human periodontal ligament fibroblasts under oxidative stress.

## 2. Materials and Methods

### 2.1. Preparation of Piceatannol

Piceatannol was kindly provided by Center for Valorization of Amazonian Bioactive Compounds—CVACBA (UFPA, Belém, Brazil). Briefly, in July of 2016, 500 g yellow passion fruit seeds (Passiflora edulis) were cleaned before drying at 80 °C for 8 h in an oven with airflow velocity of 2.0 m/s [29]. They were then defatted in a pilot scale Soxhlet for 3 h using ethyl acetate (12 reflux), dried 1 h at 80 °C and ground (0.5–0.8 mm^2^). 

#### 2.1.1. Extraction and Isolation 

Crude extract was obtained from a triple extraction in a glass flask with 90% ethanol (1:9, *w*/*w*) at 70 °C for 4 h each extraction and closed with screw cap [30] and a filtration (80 μm). The crude extract was then adsorbed onto an EXA118 macroporous resin (Resindion, Milan, Italy) [31] and then desorbed with ethanol [32]. The less polar fraction after desorption was concentrated on a rotary evaporator (Labconco, Kansas City, MO, USA) under vacuum at 40 °C for six hours and resuspended in methanol before injection at an estimated concentration of 50 mg/mL. This extract was diluted at 25 mg/mL in MeOH 50% resulting in a final volume of 48 mL and injected on a semi-preparative PLC 2020 – Gilson (Middleton, WI, USA), with flow of 5 mL/min for 28 min, volume of injection 1 mL, and elution with an isocratic mobile phase (20:80, ACN:water, *v*/*v*). The column used was a Gemini 5 m C_18_ 250 × 100 mm (Phenomenex, Torrance, CA, USA). All 48 unpolar fractions (between 16.2 and 18.2 min of elution) were unified, resulting in a volume of 480 mL, concentrated under vacuum and lyophilized (purified and dried extract, PDE).

#### 2.1.2. Purification, Identification, and Quantification

To evaluate the purity of piceatannol in PDE, the identification and quantification of piceatannol was performed by a UHPLC Thermo Scientific Ultimate (San José, CA, USA) 3000 equipped with a (LPG-3400RS) quaternary pump, a TCC-3000RS autosampler (San Jose, CA, USA), data software (Chromeleon 7.1 SR2, San Jose, CA, USA), flow cell (Thermo Fisher Scientific, Germering, Germany). The mobile phase was composed of ultrapure water (Solution A) and acetonitrile (Solution B), both acidified with formic acid (1%), and filtered through nylon membrane with 0.45 μm porosity. The column used was a Kinetex 2.6 μm C18 100 × 4.60 mm (Phenomenex, Torrance, CA, USA). The gradient was: 0–3 min, 15% B; 3–8 min, 15% to 17% B; 8–9 min, 17% to 90% B; 9–10 min, 90% B; 10–10.5 min, 90% to 15% B; 10.5–15 min, 15% B. The monitoring was set at 320 nm for quantification of piceatannol. This compound was identified by its retention time and spectral data as compared to authentic standard and was quantified using a five-point calibration curve. 

### 2.2. ORAC Assay

The antioxidant capacity of piceatannol was analyzed by the standardized method oxygen radical absorbance capacity (ORAC). The ORAC assay is based upon the inhibition of the peroxylradicalinduced oxidation initiated by thermal decomposition of azocompounds such as [2,2′-azobis(2-amidinopropane) dihydrochloride (AAPH)]. Samples (25 µL) were mixed with 250 µL of fluorescein (60 nM) and incubated for 10 min at 37 °C in the 96 wells microplate. The AAPH solution was added and the microplate was shaken. The fluorescence (λ_excitation_ = 485 nm, λ_emission_ = 520 nm) was registered each minute during 50 min. All samples were analyzed at three dilutions and the mean value was taken for ORAC as recommended by Huang et al. (2002) [33]. The quantification of the antioxidant activity was based on the calculation of the area under the curve as proposed by Cao et al. (1999) [34]. The antioxidant activity by ORAC was expressed as µmol of Trolox Equivalents (TE) per gram of FW [35].

### 2.3. Cell Studies

#### 2.3.1. Cell Culture 

Primary human periodontal ligament fibroblasts (hPLF) were obtained from patient under approval of human research ethics committee (CAEE 0121.0.073.000-11). These cells were grown in DMEM and Ham’s F12 nutrient medium (1:1) supplemented with 100 U/mL penicillin, 100 μg/mL streptomycin and 10% (*v*/*v*) of fetal bovine serum and maintained at 37 °C under 5% CO_2_. The culture medium was replaced every 48h. When reached the confluence (70% to 90% of cells) of the plate, the cells were trypsinized (Trypsin, Sigma-Aldrich, St. Louis, MO, USA).

#### 2.3.2. Cell Characterization 

For fibroblasts characterization flow cytometry analysis and indirect immunofluorescence were performed. First, an aliquot of cells was evaluated by flow cytometry using the Human MSC Analysis Kit (BD Biosciences, San Jose, CA, USA) to investigate the surface antigen expression of mesenchymal stem cells (CD90, CD73, and CD105) and a negative cocktail (CD45, CD34, CD11b, CD19, HLA-DR), in order to exclude a possible stem cell nature of the cells. Isotype-matched antibodies were used as controls to determine non-specific staining. Analysis was performed in BD FACSCanto II flow cytometer using FlowJo software (BD Biosciences, San Jose, CA, USA). For indirect immunofluorescence the cells were seeded at a density of 1 × 10^5^ cells/well glass coverslips in 24-well plates and this process involved the following steps: fixation in 2% paraformaldehyde for 10 min; washing with PBS (phosphate buffered saline); membrane permeabilization with 0.5% Triton X-100 (Sigma^®^, St. Louis, MO, USA) solution for 15 min; washing with PBS; incubation in 1% PBS/BSA (BSA, Bovine Serum Albumin, Sigma^®^, St. Louis, MO, USA) for 30 min; incubation with the primary monoclonal antibodies diluted in 1% PBS/BSA for at least 12 h and at most 18 h in a humid chamber at 4 °C. The primary antibodies used were: anti-vimentin, mouse monoclonal (1:100; Diagnostic BioSystems, Pleasanton, CA, USA); anti-cytokeratin AE1/AE3, mouse monoclonal (1:100; Invitrogen Molecular Probes, Eugene, OR, USA) and anti-fibronectin, rabbit monoclonal (1:100; Dakocytomation, Glostrup, KO, Denmark). For the detection of the primary antibody, incubation was performed in solution containing the secondary antibody conjugated to AlexaFluor 488 or 588 (Invitrogen^®^, Carlsbad, CA, USA) for 1 h in a dark humid chamber at room temperature. The nuclei were labeled with Hoechst 33258 (1: 2000, Sigma^®^, St. Louis, MO, USA). The coverslips were then immersed in PBS and distilled water and mounted with ProLong^®^ Gold antifade reagent (Invitrogen^®^, Carlsbad, CA, USA). Afterwards, they were analyzed in a fluorescence microscope (AxioScope.A1, Zeiss^®^, Oberkochen, Germany), equipped with a digital still camera (AxiocamMRc, Zeiss^®^, Oberkochen, Germany). As a negative control, the same protocol was performed without incubation of the primary antibody.

#### 2.3.3. Oxidative Stress Induction

To mimic a periodontitis cell injury, we have stimulated hPFL with 200 µM H_2_O_2_ for 1 h in combination or not with different concentrations of piceatanol (0.1 to 20µM) [22,36].

#### 2.3.4. Cell Viability and General Metabolism Status 

hPLF were seeded at a density of 2 × 10^4^ cells/well in 96-well culture plates and incubated for 24 h on 5% CO_2_ and 37 °C. After that, the solution containing H_2_O_2_, piceatannol or a combination of both were prepared 20 min before treatment and added to each well according to the experimental group. Cell viability was determined by Trypan blue dye exclusion test [37] and general metabolism status was determined by the colorimetric MTT reduction assay [38]. In viability assay, an aliquot of fibroblasts suspension was mixed with trypan blue (0.4%) and visually examined to determine exclusion dye by the viable cells. For general metabolism assay, the fibroblasts were incubated with 100 µL of MTT (5 mg/mL) after cells being treated with piceatannol as previously described. After 2.5 h of incubation at 37 °C, the MTT solution was removed, and the resulting formazan crystals formed in viable cells were solubilized with DMSO. Absorbance values were read at 570 nm on a microplate reader (Glomax^®^ multi detection system—Promega Corporation, Madison, WI, USA). As cells varied their metabolism according to exposure agents and may be related to the dose or concentration of the compounds, MTT assay values may not reflect the direct increase or decrease of viable cells in the treatments. On this way, general metabolism status was expressed as MTT absorbance/viable cells [39,40]. All analysis was performed in triplicate and vehicle (methanol 20%) was also tested. 

#### 2.3.5. Cell Membrane Integrity and ATP Production

To proceed with all the subsequent analyses of oxidative stress two different concentrations of piceatannol were chose based on cell viability and general metabolism results. hPLF were seeded at a density of 2 × 10^4^ cells/well in 96-well culture plates and after 24 h, they were treated during 1 h with 0.1 and 1 µM of piceatannol in the presence or absence of H_2_O_2_ (200 μM). The membrane integrity and ATP production were analyzed through the mitochondrial tox-glo™ Assay (Promega, Madison, WI, USA) according to the manufacturer’s instruction. Cells were incubated with cytotoxicity reagent at 37 °C for 30 min and fluorescence was measured at 520 nm (Glomax^®^ multi detection system— Promega Corporation, Madison, WI, USA). This value represents the membrane integrity (MI). Thereafter the same plate was equilibrated at room temperature and then incubated with the ATP detection substrate; luminescence was measured after 5 min to 1 h for the detection of ATP production (Glomax^®^ multi detection system—Promega Corporation, Madison, WI, USA). All analysis was performed in triplicate and vehicle (methanol 20%) was also tested.

#### 2.3.6. Total Antioxidant Capacity

The total antioxidant capacity was determined according to the Trolox equivalent antioxidant capacity (TEAC). Trolox (6-hydroxy-2,5,7,8-tetramethylchroman-2-carboxylic acid) is a potent water-soluble analogue of vitamin E. In this assay, hPLF were seeded at a density of 1 × 10^5^ cells/well in 24-well culture plates, after 24 h they were treated with 0.1 and 1 µM piceatannol for 1 h in the presence or absence of H_2_O_2_ (200 μM). Following that, 2,2′-azino-bis(3-ethylbenzothiazoline-6-sulphonate) (ABTS™) was incubated with persulfate to produce the blue-green ABTS^+^. Antioxidants present in the sample cause a reduction in absorption proportional to their concentration. The antioxidant capacities (TEAC) of the samples are expressed as mmol/L/viable cells using a calibration curve plotted with different amounts of Trolox, and their absorbance measured at 740 nm [41]. All analysis was performed in triplicate and vehicle (methanol 20%) was also tested.

#### 2.3.7. Reduced Glutathione

The reduced glutathione (GSH) was measured using the GSH/GSSG-Glo™ Assay (Promega, Madison, WI, USA) according to the manufacturer´s instruction. After 1-h treatment hPLF (2 × 10^4^ cells/well; 96 well plate) with 0.1 and 1 µM piceatannol for 1 h in the presence or absence of H_2_O_2_ (200 μM), medium was removed and cells lyses performed using total glutathione reagent. After five minutes, cells were incubated with Luciferin generation reagent for 30 min and then Luciferin detection reagent was added. After 15 min, luminescence was measured. All analysis was performed in triplicate and vehicle (methanol 20%) was also tested.

#### 2.3.8. Statistical Analysis

The results were expressed as mean ± standard error and analyses were performed using one-way variance and Tukey post-test. ANOVA assumptions (data normality through Shapiro–Wilk test and homogeneity of variances) were previously verified. Data were shown as median ± standard deviation when *p* < 0.05. Bioestat 5.0 and Graph Pad Prism 5 (GSL Biotech LLC, San Diego, CA, USA) were used for data analysis.

## 3. Results and Discussion

Phytotherapeutic formulations have been used as adjunctive therapy with significant improvement in periodontal diseases [27]. Here we evaluate the effect of Piceatannol in periodontal diseases. Piceatannol is a naturally phenolic compound found in a variety of plant sources including grapes, rhubarb, peanuts, sugarcane, white tea, and the seeds of passion fruit (*Passiflora edulis*). Its effects have been investigated extensively in the past decade. Several biological functions have been reported as antioxidative, anti-inflammatory, anticancer, antidiabetic, cardioprotective, neuroprotective, and immunomodulatory properties [42]. The beneficial effects of piceatannol as antioxidant agent on periodontal diseases were not previously described. 

Initially, in the present study piceatannol was obtained from yellow passion fruit seeds (*Passiflora edulis*). The final concentration of 0.0425 g of piceatannol was found per gram of dried yellow passion fruit seeds. This high concentration is in agreement with a recently reported study (0.0368 g/g dried seeds) [43]. Purity of piceatannol in purified and dried extract (PDE) achieved 66.4%. After, we tested the piceatannol antioxidant capacity by the standardized method oxygen radical absorbance capacity (ORAC) assay and the results indicated that piceatannol analyzed had 6739 µmol Trolox equivalent/mL of a 1 mM piceatannol confirming the high antioxidant capacity of this stilbene. Once the antioxidant capacity of the piceatannol was, the ability for it to protect against oxidative stress was tested in the fibroblast of the human periodontal ligament cells. 

**Figure 1:**
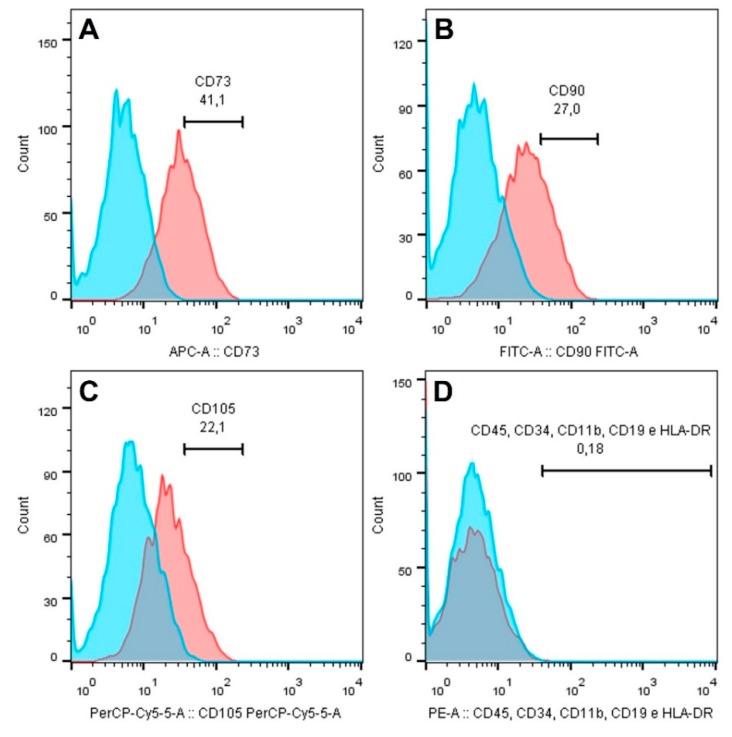
Expression of stem cell markers CD90 (**A**), CD73 (**B**), CD105 (**C**), and negative cocktail (**D**) in fibroblasts of periodontal ligament.

**Figure 2:**
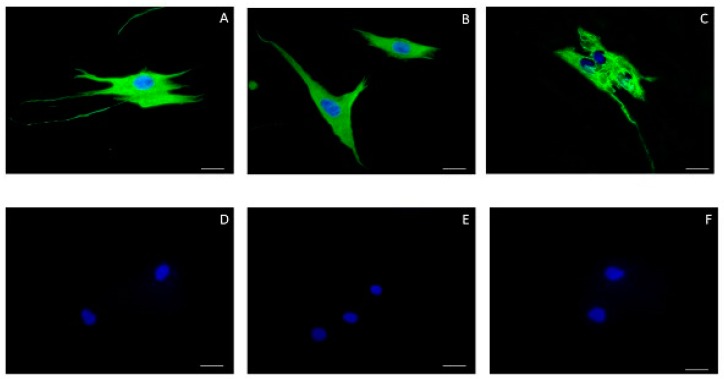
Indirect immunofluorescence. Fibroblasts of periodontal ligament expressed vimentin (**A**) and fibronectin (**B**), but no cytokeratin AE1/AE3 (**C**). Negative controls were shown on (**D**–**F**). Nuclear staining was performed with Hoechst. Scale bar = 20 μm.

Fibroblasts of the human periodontal ligament are an important cell population responsible for the maintenance of the periodontal ligament integrity and consequent fixation of the dental element to the alveolar bone [44]. The periodontal ligament is a loose connective tissue formed by several cellular populations, the fibroblast being the most numerous, and also containing a large amount of collagen fibers produced by it immersed in the extracellular matrix [45]. To exclude the possibility of the cells isolated in this study were periodontal ligament stem cells, immunophenotyping was performed for mesenchymal stem cell markers. The data of flow cytometry analysis demonstrated a low percentage of cells positive for the surface markers CD73 (41.1%), CD90 (27.9%), and CD105 (22.1%), while only 0.18% of cells were positive for CD45, CD34, CD11b, CD19, and HLA-DR (Figure 1). To confirm that the cells were fibroblasts, indirect immunofluorescence was performed to stain target antigens in this cell population. Vimentin and fibronectin proteins underwent immunostaining confirming the cells were fibroblasts. There was no labeling for cytokeratin, which excluded the possibility of being epithelial cell origin (Figure 2).

In order to simulate fibroblast injury by inducing oxidative stress to simulate periodontal disease, we chose hydrogen peroxide (H_2_O_2_) that has been reported in other studies as capable to easily translocate cell membranes and generate hydroxyl radicals [46]. In our study, hPLF were exposed to 200 µM H_2_O_2_ for 1 h and injuries related to this exposure were evaluated after combined maintenance of these cells in a solution containing piceatannol at different concentrations. It is important to note that the carrier substance used to dissolve piceatannol (methanol 20%) was tested in all parameters (viability and general metabolism status, membrane integrity and amount of ATP, TEAC and reduced glutathione) at maximum volume necessary to prepare our most concentrated exposure solution (20 µM). No statistical difference was observed when compared to the control.

**Figure 3:**
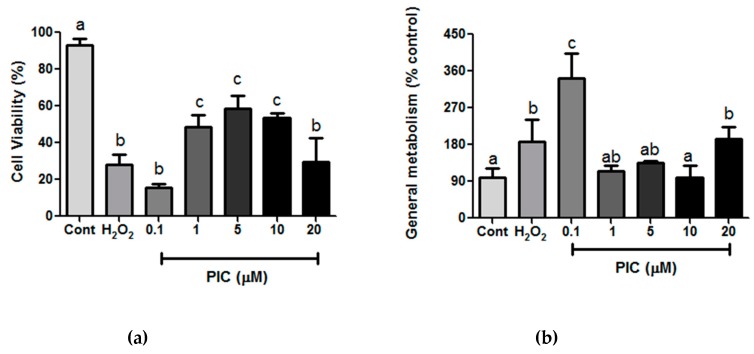
(**a**) Cell viability and (**b**) general metabolism status after 1-h treatment with hydrogen peroxide (H_2_O_2_) (200 µM) and different concentrations of piceatannol (Pic); Cont: control. The results are expressed as mean ± SD. Statistical differences between treatments at the same analysis were shown by lower case (a–c) on graph (*p* ≤ 0.05).

As expected, hPLF exposed to H_2_O_2_ had a significant decrease of cell viability in 32 ± 12% when compared to control (Figure 3a). This condition was used to evaluate whether piceatannol in different concentrations (0.1–20 μM) could protect cell from death and act directly on cell viability. We showed that piceatannol at concentrations of 1, 5, and 10 μM maintained cell viability of hPLF in 48.5% (±6.6), 58.3% (±7.2), and 60%(±13.9), respectively (Figure 3a), indicating a direct effect on cell protection and confirming previous study under the same range concentration (1–10 μM) in the presence of amyloid β-peptides (Aβ) as a inducer of toxicity [47]. In this study, authors have showed that piceatannol had the ability to increase cell viability up to 60% and 79% when compared to control group. In this way, piceatannol was one of the most effective of stilbenes tested on PC12 cells. 

On the other hand, the concentrations of 0.1 µM and 20 μM of piceatannol showed significant decrease of cell viability related to control, but no difference to H_2_O_2_ exposed cells. One possible explanation could be the dual effect of piceatannol. The pro- and antioxidant activity of piceatannol has already been tested in several models of cell culture [48,49] suggesting that the piceatannol effect depends on the type of cell, its metabolic activity and phase of the cell cycle analyzed [44]. In a study with leukemic cells, piceatannol showed potent antioxidant capacity against DNA damage. Its protective activity was 58–58.4% between 1.25 and 5 μM with pro-antioxidant effect between 10 and 50 μM [49]. The same was observed on astrogliomas cell culture model, with pro-antioxidant effect at concentrations above 20 μM and a cytoprotective capacity at lower concentrations (5 and 10 μM) [48]. In another study, the piceatannol effect on restoring the endothelial cell activity was evaluated under high-glucose oxidative stress. Interestingly, the piceatannol at 0.01 μM and 0.1 μM did not induce cell death for a 24 h exposure period and was also able to improve DDAH activity and thiol concentration [50]. These differences between 0.1 μM PIC effect could be explained by the difference on oxidative stress induction by peroxide and high-glucose, but additional studies are required to clarify that. 

We also checked the piceatannol-induced effect on the general metabolism status of hPLF. The enzymatic reduction of MTT to formazan served to assess the general metabolism status at the same concentration of H_2_O_2_ and piceatannol used in cell viability assay. Our result showed that the metabolism at 1, 5, and 10 μM of piceatannol remained similar to the control while only 0.1 μM caused a higher metabolism compared to control and H_2_O_2_ (Figure 3b). The metabolism could be closely linked to cell viability, proliferation, and oxidative stress [51]. In this way, in order to understand how piceatannol could affect hPLF the concentrations of 0.1 and 1 μM piceatannol were chosen to continue the study analysis.

**Figure 4:**
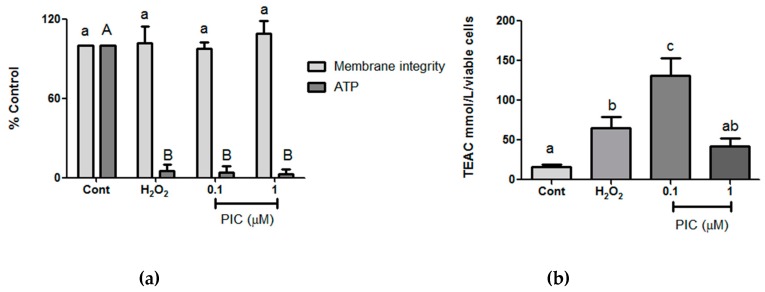
(**a**) Cell membrane integrity and ATP production and (**b**) total antioxidation capacity after 1-h treatment with hydrogen peroxide (H_2_O_2_) (200 µM) and different concentrations of piceatannol (Pic). The results are expressed as mean ± SD. Statistical differences between treatments at the same analysis were shown by lower case (a–c) and upper case (A–B) on graph (*p* ≤ 0.05).

We next checked the cell membrane integrity and ATP production (Figure 4a). Regarding membrane integrity, our results showed no difference among groups, suggesting that cell death observed in our first test likely occurred by apoptosis since the protease used in this assay is an indicative of cell death by necrosis. Besides that, piceatannol did not protect cells from mitochondrial damage as measured by decrease of ATP production in exposed H_2_O_2_ and piceatannol groups. This can be explained by the ability of stilbenes to inhibit the ATPase activity of ATP synthase, an enzyme that catalyzes the synthesis of ATP molecules by the oxidative phosphorylation process [52].

In order to evaluate biochemistry parameters related to antioxidant defenses in the hPLF under oxidative stress conditions, Trolox equivalent antioxidant capacity (TEAC) and reduced glutathione (GSH) levels were assessed. The concentration of 0.1 μM of piceatannol showed the highest TEAC value, whereas the concentration of 1 μM was not statistically different in relation to the control and peroxide groups (Figure 4b).

**Figure 5:**
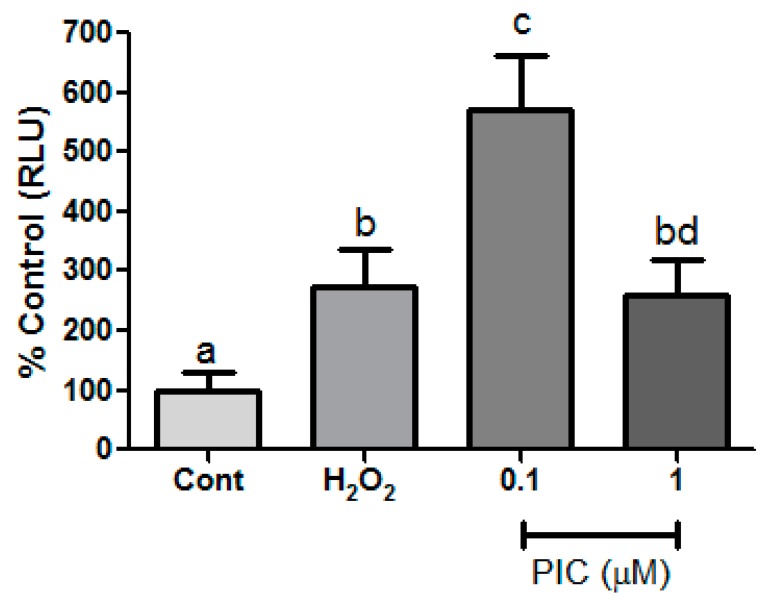
Reduced glutathione was measured after 1-h treatment with hydrogen peroxide (H_2_O_2_) (200 µM) and different concentrations of piceatannol (PIC). The results are expressed as mean ± SD. Statistical differences between treatments at the same analysis were shown by lower case (a–d) on graph (*p* ≤ 0.05).

The results of GSH, the most important non-enzymatic cellular antioxidant defense [53] were similar to the results of the TEAC (Figure 5). A study showed that dose-dependent piceatannol increases the concentration of GSH in keratinocytes but without any induction of injury [53]. In another study, the intracellular level of GSH was evaluated on melanoma cell (B16 cells). Authors have showed a concentration-dependent increase of GSH levels by 5, 10 and 50 µM [54]. This increase could be possible a result from directly action of piceatannol on enzymes that synthesize or suppress GSH degradation. In the present study, the increasing GSH at 0.1 μM indicates that the cell is activating more mechanisms of damage control, which is not enough to maintain cell viability. On the other hand, the fact that the concentration of 1 μM was not different from peroxide, suggests that the maintenance of the cellular viability promoted by piceatannol does not depend on the action of GSH and probably another antioxidant route. 

Herbal extracts from natural products are considered potential candidates for the treatment of chronic periodontitis, substantially increasing the number of in vitro and in vivo studies related to the efficacy of medicinal plants with known anti-inflammatory and antibacterial actions [55,56]. In our study, the effects of piceatannol on hPLF under mimetic effect of inflammatory conditions was concentration-dependent. It was possible to observe the modulation of antioxidant defenses at the concentration of 0.1 μM piceatannol through the increase of TEAC and GSH levels, even though it was not able to maintain cell viability. However, 1 μM of piceatannol was able to maintain cell viability, but did not modulated the antioxidant response. Further studies should be conducted to better understand the mechanisms of antioxidant action of piceatannol in periodontal ligament fibroblasts. We suggest that other parameters of oxidative stress should be analyzed beside the cellular physiology through maintaining the collagen production capacity of the fibroblasts.

## 4. Conclusions

In conclusion, our study brings evidence that piceatannol is a promising new antioxidant herbal extract that could be helpful to prevent human periodontal ligament injury under oxidative stress in an in vitro model of periodontitis.

## Abbreviations

ROS	reactive oxygen species
MeOH	methanol
PDE	purified and dryed extract
PLC	personal liquid chromatography system
UHPLC	Ultra high performance liquid chromatography
PBS	phosphate buffer solution
hPLF	human periodontal ligament fibroblasts
MTT	3-(4,5-dimethylthiazol-2-yl)-2,5-diphenyltetrazolium bromide
DMSO	dimethyl sulfoxide
TEAC	trolox equivalent antioxidant capacity
GSH	reduced gluthatione
ORAC	oxygen radical absorbance capacity
